# MCMV-based vaccine vectors expressing full-length viral proteins provide long-term humoral immune protection upon a single-shot vaccination

**DOI:** 10.1038/s41423-021-00814-5

**Published:** 2022-01-07

**Authors:** Yeonsu Kim, Xiaoyan Zheng, Kathrin Eschke, M. Zeeshan Chaudhry, Federico Bertoglio, Adriana Tomić, Astrid Krmpotić, Markus Hoffmann, Yotam Bar-On, Julia Boehme, Dunja Bruder, Thomas Ebensen, Linda Brunotte, Stephan Ludwig, Martin Messerle, Carlos Guzman, Ofer Mandelboim, Michael Hust, Stefan Pöhlmann, Stipan Jonjić, Luka Čičin-Šain

**Affiliations:** 1 Department of Viral Immunology, Helmholtz Centre for Infection Research (HZI), Braunschweig, Germany; 2 Department of Biotechnology, Institut für Biochemie, Biotechnologie und Bioinformatik, Technischen Universität Braunschweig, Braunschweig, Germany; 3 Oxford Vaccine Group, University of Oxford, Oxford, UK; 4 Center for Proteomics, University of Rijeka, Faculty of Medicine, Rijeka, Croatia; 5 Infection Biology Unit, German Primate Center, Göttingen, Germany; 6 Faculty of Biology and Psychology, Georg-August-University Göttingen, Göttingen, Germany; 7 Department of Immunology, Rappaport Faculty of Medicine, Technion - Israel Institute of Technology, Haifa, Israel; 8 Infection Immunology, Institute for Medical Microbiology and Hospital Hygiene, Health Campus Immunology, Infectiology and Inflammation, Otto-von-Guericke-University Magdeburg, Magdeburg, Germany; Immune Regulation, Helmholtz Centre for Infection Research, Braunschweig, Germany; 9 Department of Vaccinology and Applied Microbiology, Helmholtz Centre for Infection Research, Braunschweig, Germany; 10 Institute of Virology, Medical University Münster, Münster, Germany; 11 Institute of Virology, Hannover Medical School (MHH), Hannover, Germany; 12 German Centre for Infection Research (DZIF), Hannover-Braunschweig Site, Hannover, Germany; 13 Centre for Individualized Infection medicine (CiiM), a joint venture of HZI and MHH, Hannover, Germany

**Keywords:** Vaccine vector, SARS-CoV-2, Influenza, Cytomegalovirus, humoral imunity, Vaccines, Vaccines, Immunological memory

## Abstract

Global pandemics caused by influenza or coronaviruses cause severe disruptions to public health and lead to high morbidity and mortality. There remains a medical need for vaccines against these pathogens. CMV (cytomegalovirus) is a β-herpesvirus that induces uniquely robust immune responses in which remarkably large populations of antigen-specific CD8^+^ T cells are maintained for a lifetime. Hence, CMV has been proposed and investigated as a novel vaccine vector for expressing antigenic peptides or proteins to elicit protective cellular immune responses against numerous pathogens. We generated two recombinant murine CMV (MCMV) vaccine vectors expressing hemagglutinin (HA) of influenza A virus (MCMV^HA^) or the spike protein of severe acute respiratory syndrome coronavirus 2 (MCMV^S^). A single injection of MCMVs expressing either viral protein induced potent neutralizing antibody responses, which strengthened over time. Importantly, MCMV^HA^-vaccinated mice were protected from illness following challenge with the influenza virus, and we excluded that this protection was due to the effects of memory T cells. Conclusively, we show here that MCMV vectors induce not only long-term cellular immunity but also humoral responses that provide long-term immune protection against clinically relevant respiratory pathogens.

## Introduction

Severe acute respiratory syndrome coronavirus 2 (SARS-CoV-2) and influenza A virus (IAV) are well-known viruses with a zoonotic origin that have caused global pandemics with severe consequences on human health and economies. SARS-CoV-2, which caused the coronavirus disease 2019 (COVID-19) pandemic, was first identified in late 2019 in Wuhan, China. The severe COVID-19 global pandemic has claimed millions of lives and resulted in severe economic disruption worldwide. Influenza pandemics have also resulted in global disruptions, such as the H1N1 Spanish flu in 1918, the H3N2 Hong Kong flu in 1968 and the H1N1dpm09 swine flu in 2009, and resulted in rapid global spread of this respiratory disease. In addition to these influenza pandemics, seasonal influenza epidemics regularly cause elevated morbidity and mortality in the colder seasons. Both IAV and SARS CoV-2 may cause mild to severe respiratory illnesses and pose a particular threat to at-risk groups, such as elderly people or people with pre-existing medical conditions. Both of these respiratory viruses depend on a viral surface protein for attachment and entry into host cells. In the case of IAV, viral hemagglutinin (HA) is the major surface glycoprotein required for cell entry [1, 2]. Likewise, SARS-CoV-2 uses the spike protein (S) to bind its cellular receptor ACE2 and to drive membrane fusion during virus entry [3–6]. Therefore, SARS-CoV-2 S and IAV HA are the main antigenic targets in vaccine formulations against these viruses.

Numerous efforts are underway to counter COVID-19. There are more than 200 vaccine projects targeting SARS-CoV-2 [7] using formulations that include viral proteins, viral vector vaccines, and mRNA vaccines. Some of these vaccines have already been approved for use in humans or are in advanced clinical trials with promising results. However, all of the candidates raise safety concerns due to side effects such as fever, fatigue and headache [8], and most vaccines (or vaccine candidates) require a prime/boost vaccination protocol at multiple-week intervals, raising issues of delivery logistics and compliance. Although mRNA vaccines show great promise in the context of the COVID-19 pandemic [9], experience with their use in clinical settings remains limited [10]. Vaccines against influenza target the predicted prevailing strains in each upcoming flu season and are especially recommended for people at high risk, such as children, elderly individuals and immunocompromised individuals [11]. While influenza vaccines are available, their efficacy is approximately 19-60% depending on the flu season [12, 13].

Viral vectors do not need adjuvants because they contain molecular patterns recognized by innate immune receptors and naturally induce both the cellular and humoral branches of the adaptive immune response [14, 15]. Therefore, they have been developed by numerous research laboratories using a variety of viral vectors, including poxviruses, adenoviruses and herpesviruses [16–25]. Among them, cytomegalovirus (CMV) is a highly promising platform for vaccine design, with several advantages and unique features. CMV infection is usually asymptomatic, but the virus persists for life, inducing a strong and durable inflationary CD8^+^ T-cell response [26–32]. The optimal design of CMV-based vaccines is an area of intensive study. Numerous studies on CMV vaccines have indicated that powerful CD8^+^ T-cell responses can be induced by CMV infection. Various experimental CMV vectors expressing single epitopes against diverse pathogens provide immune protection based on a robust epitope-specific CD8^+^ T-cell response [18, 20, 21, 28, 33–37]. In alignment with this strategy, boosting or maintaining strong CD8^+^ T-cell populations but diminishing viral pathogenesis is another focus of CMV vaccine vector design [38–42]. Interestingly, an MCMV vector encoding a CD8^+^ T-cell epitope derived from the IAV HA gene [43] has been found to induce protective CD8^+^ T-cell responses against IAV, but only when administered intranasally and eliciting responses from mucosa-resident CD8^+^ T cells [37]. These effects are similar to effects observed upon immunization with an MCMV vector targeting an epitope of the respiratory syncytial virus [44].

In this study, we constructed recombinant MCMVs expressing either the full-length IAV HA protein (MCMV^HA^) or SARS-CoV-2 S protein (MCMV^S^). We used these vectors to immunize mice and analyzed their immunoprotective effects. We also compared MCMV^HA^ immune protection to that induced by a vector expressing the optimally positioned immunodominant epitope from the same virus. We found that immunization with MCMVs expressing a full-length protein efficiently induced neutralizing antibodies and protected the animals against viral challenge despite poor CD8^+^ T-cell responses. Experiments in B-cell-deficient JHT mice demonstrated that the immune protection conferred by single-dose administration of the MCMV vector was not only robust and lasting but also antibody-dependent. This advances the design of MCMV-based vaccines.

## Materials and methods

### Ethics statement

BALB/cJRj and C57BL/6JRj mice were purchased from commercial vendors (Janvier, Le Genest Saint Isle, France). B6.129P2-*Igh*-J^tm1Cgn^/J (JHT) mice were bred in the animal facility of Helmholtz Center for Infection Research, Braunschweig. The animals were housed under SPF conditions at HZI or Hebrew University in Jerusalem and handled according to good animal practice as defined by the Federation of Laboratory Animal Science Associations (FELASA). The animal experiments were approved by the Lower Saxony State Office of Consumer Protection and Food Safety and the Hebrew University Medical School Ethics committee.

### Cell culture and viruses

Vero E6 (CRL-1586), Vero76 (ATCC CRL-1586), 293 T (DSMZ ACC-635), MDCK (CCL-34) and M2-10B4 cells (ATCC CRL-1972) were cultured in DMEM (Gibco, NY, USA) supplemented with 10% fetal bovine serum (FBS), 2 mM L-glutamine, 100 IU/mL penicillin and 100 μg/mL streptomycin. C57BL/6 primary mouse embryonic fibroblasts (MEFs) were prepared in-house from C57BL/6JRj mice. The PR8M variant of influenza A/PR/8/34 was obtained from the strain collection at the Institute of Molecular Virology, Muenster, Germany. The SARS-CoV-2 South Tyrol strain (FI strain, hCoV-19/Germany/Muenster_FI1103201/2020, GISAID database ID: EPI_ISL_463008) was isolated by the Stephan Ludwig laboratory. MCMV^WT^ refers to the BAC-derived molecular clone (pSM3fr-MCK-2fl clone 3.3) [45].

### Virus mutagenesis

MCMV virus mutants were created based on the BAC molecular clone pSM3fr-MCK-2fl clone 3.3; recombinant variants were generated by en passant mutagenesis, as described previously [46, 47]. The construction of MCMV^IVL^ has been described previously [37].

MCMV variants expressing the hemagglutinin protein were constructed using either the wild-type MCMV genome or the Δm152-RAE1γ MCMV genome [39] to generate MCMV^HA^ and Rae-1γMCMV^HA^. MCMV^HA^ was generated by replacing the m157 ORF with an expression cassette containing the human CMV major immediate-early enhancer and promotor (hMIEP) and the hemagglutinin ORF (Fig. S4A). The hemagglutinin ORF was amplified from the pUC18 vector containing the hemagglutinin ORF of the PR8 strain (UniProt P03452), which was kindly provided by Peter Stäheli. Recombinant MCMV expressing the SARS-CoV-2 spike was generated by inserting the full-length spike ORF in place of the ie2 gene by replacing the start and stop codon of the ie2 ORF with the start and stop codon of the spike ORF. The spike ORF was amplified from the pCG1-SARS-2-S plasmid [4, 6] that harbors the spike ORF from the Wuhan-1 strain (GenBank: MN_908947).

### Virus stock generation and plaque assay

BAC-derived mutant MCMVs were propagated in M2-10B4 cells and purified by sucrose density gradient as described previously [47]. Influenza virus was generated, and infectious virus was quantified as described previously [37]. SARS-CoV-2 virus stocks were generated essentially as described previously [48]. Briefly, infected Vero E6 cells and supernatants were harvested, centrifuged to remove the cell debris and concentrated with Vivaspin® 20 concentrators (Sartorius, Goettingen, Germany) according to the manufacturer’s instructions. The infectious titer was determined by serially diluting the virus stocks and then infecting Vero E6 cell monolayers in 24-well plates for an hour at 37 °C. Thereupon, the inoculum was removed, and the cells were overlaid with 1.5% methylcellulose. The cells were incubated at 37 °C for 4 days, fixed with 6% PFA for an hour, and stained with crystal violet, and the plaques were counted under an inverted microscope.

### Virus infection in vivo

Female BALB/c mice aged 7–8 weeks were intraperitoneally (i.p.) immunized with 2 × 10^5^ PFU of recombinant MCMVs expressing antigens or with parental control virus diluted in PBS (200 µl per animal). Blood was acquired at the indicated time points.

For B-cell-deficient animal challenge experiments, 6- to 8-week-old BALB/c, C57BL/6 and JHT female mice were i.p. immunized with 2 × 10^5^ PFU of MCMV^HA^ or MCMV^IVL^ diluted in PBS. For influenza infection, mice were first anesthetized with ketamine (10 mg/ml) and xylazine (1 mg/ml) in 0.9% NaCl (100 μl/10 g body weight) and then challenged intranasally with 1100 FFU of PR8M influenza virus as described previously [37].

Rae-1γMCMV^HA^ immunizations were performed by infecting C57BL/6 mice f.p. with 2 × 10^5^ PFU of the indicated recombinant MCMVs. Twenty-one days post-immunization, the mice were challenged i.n. with either a high (100 hemagglutinin units, HU) or low dose (40 HU) of the PR8M influenza virus.

### Detection of anti-spike antibodies in mouse sera

ELISA (enzyme-linked immunosorbent assay) was used to detect SARS-CoV-2 spike-specific IgGs in mouse sera. Antigens were produced in insect cells using a baculovirus-free system according to previous publications [49] and immobilized (30 ng/well) in carbonate buffer (50 mM NaHCO_3_/Na_2_CO_3_, pH 9.6) at 4 °C overnight. The ELISA plates were blocked with 2% (w/v) milk powder and 0.05% Tween-20 in PBS (2% MBPST) and washed with 0.05% Tween-20 in H_2_O. To determine the IgG titers, mouse sera were diluted 1:100 in 2% MBPST and further titrated via ELISA using S1-S2-His and RBD-SD1-HIS as antigens or BSA as a control for nonspecific binding. In addition, all sera were also tested at the highest concentration for nonspecific cross-reactivity on Expi293F cell lysates and lysozymes (both 30 ng/well). After 1 h of incubation at 37 °C and washing as reported above, the mouse IgGs were detected using goat anti-mouse serum conjugated with horseradish peroxidase (HRP) (Sigma–Aldrich, Munich, Germany). The bound antibodies were visualized with tetramethylbenzidine (TMB) substrate. After stopping the reaction by the addition of 5% H_2_SO_4_, the absorbance at 450 nm with the 620 nm reference absorbance subtracted was measured in an ELISA plate reader (Epoch, BioTek, Winooski, VT, USA). Titration assays were performed using 384-well microtiter plates (Greiner, Kremsmuenster, Austria) using a Precision XS microplate sample processor (BioTek, Winooski, VT, USA), EL406 washer dispenser (BioTek, Winooski, VT, USA) and BioStack Microplate stacker (BioTek, Winooski, VT, USA). The EC_50_ values were calculated with GraphPad Prism Version 6.1 via fitting to a four-parameter logistic curve.

### Antibody avidity assay

Antibody avidity determination was performed essentially as reported previously [50]. ELISA 96-well plates (Costar, Corning, NY, USA) were used to immobilize the SARS-CoV-2 S1-S2-HIS ectodomain at 100 ng/well in carbonate buffer (50 mM NaHCO_3_/Na_2_CO_3_, pH 9.6) at 4 °C overnight. After blocking in 2% MPBST, mouse sera were pooled to a final dilution of 1:300 in 2% MPBST and incubated for 1 h at 37 °C on the immobilized S1-S2-HIS. After washing with PBS with 0.05% Tween 20 (v/v) (PBST), the plates were incubated with the indicated dilutions of NaSCN for 15 min at room temperature and 350 rpm and then immediately washed with PBST. IgGs were detected using anti-mouse serum conjugated with HRP (A0168, Sigma–Aldrich, Munich, Germany), and the absorbance was measured as described above. The values obtained in the absence of NaSCN were normalized to represent 100% IgG binding. Hence, the avidity of spike-specific antibodies was calculated from the ratio of the absorbance of antibodies bound after treatment with graded concentrations of NaSCN relative to the signal in the absence of NaSCN. One-way ANOVA to compare multiple groups was performed with Dunnett’s correction for multiple analyses.

### Hemagglutination inhibition assay (HAI)

Serum samples were derived from mice at Day 28 post-MCMV^HA^ or MCMV^IVL^ immunization (dpi) or at Day 5 after IAV challenge. Sera were tested for HA-specific antibody titers by standard methods using a 0.7% v/v turkey erythrocyte suspension, as described previously [51]. In brief, to remove nonspecific inhibitors, the sera were treated with 1∶5 and 1∶2 receptor-destroying enzymes (Seiken, Tokyo, Japan) overnight before heat inactivation (56 °C, 30 min). The serum samples were added to 96-well v-bottomed microtiter plates at a starting dilution of 1:10. The serum HI titers are expressed as the reciprocal of the highest dilution at which 50% hemagglutination was inhibited. A surrogate correlate of protection was extrapolated from seasonal vaccination in humans using a titer above ≥40 to indicate seroprotection and a serum titer less than 5 as a negative result.

### In vitro serum neutralization titer (SNT) assay

Serum was heat-inactivated for 30 min at 56 °C, serially diluted in 1:2 steps and incubated with 100 PFU/100 µl of SARS-CoV-2 for an hour at RT. For IgM depletion, heat-inactivated sera were incubated with 2-ME for an hour at RT prior to mixing with the virus. Vero-E6 cells (2 × 10^4^) seeded in 96-well plates were inoculated with serum and virus and incubated at 37 °C and 5% CO_2_ for 1 h. After removing the inoculum, the cells were overlaid with 1.5% methylcellulose. The infected cells were incubated at 37 °C and 5% CO_2_ for 3 days prior to crystal violet staining and plaque counting. The serum titer resulting in a 50% reduction in plaques (VNT_50_) was assessed.

### Pseudovirus neutralization assay

We used vesicular stomatitis virus (VSV) pseudotyped with SARS-CoV-2 S according to a published protocol [52] and as described in detail previously [53]. In brief, 293 T cells were transfected with expression plasmids for SARS-CoV-2 S protein of Wuhan/Hu-1/2019 (lineage B, with the D614G mutation inserted, codon-optimized), hCoV-19/England/MILK-9E05B3/2020 (lineage B.1.1.7, codon-optimized), or hCoV-19/South Africa/NHLS-UCT-GS-1067/2020 (lineage B.1.351, codon-optimized) or an empty expression vector (negative control, used to generate bald control particles) by the calcium-phosphate method. At 24 h post-transfection, the transfection medium was removed, and cells were inoculated with a replication-deficient VSV vector lacking genetic information for VSV glycoprotein (VSV-G) and instead coding for an enhanced green fluorescent protein and firefly luciferase from independent transcription units, VSV*ΔG-FLuc (kindly provided by Gert Zimmer, Institute of Virology and Immunology, Mittelhäusern, Switzerland) [54]. Following 1 h of incubation at 37 °C and 5% CO_2_, the inoculum was removed, and the cells were washed with PBS. Then, culture medium containing anti-VSV-G antibody (culture supernatant from I1-hybridoma cells; ATCC CRL-2700) was added, and the cells were further incubated. The pseudotype particles were harvested at 16-18 h post-inoculation. For this, the culture medium was collected and centrifuged (2,000 x g, 10 min, RT) to pellet cellular debris, and the clarified supernatant was transferred into fresh tubes and stored at −80 °C until further use. Each batch of pseudotypes was pretested for comparable transduction efficiencies by the respective S proteins and absence of transduction by control particles lacking any surface glycoprotein before being used in neutralization experiments.

For neutralization experiments, equal volumes of pseudotype particles and serum dilution or medium (control) were mixed and incubated for 30 min at 37 °C before being inoculated onto Vero76 cells grown in 96-well plates (100 µl/well; the samples were analyzed in technical triplicates). Transduction efficiency was analyzed at 16 h post-transduction. For this, the medium was aspirated, and cells were lysed by incubation with Cell Culture Lysis Reagent (Promega, Madison, WI, USA) for 30 min. The lysates were transferred to white 96-well plates, and luciferase activity was measured by adding a commercial substrate (Beetle Juice, PJK, Kleinblittersdorf, Germany) and recording the luminescence signals (given as counts per second) with a Hidex Sense plate luminometer (Hidex, Okegawa, Victoria).

### Influenza virus ex vivo titration

Mice were sacrificed by CO_2_ inhalation. The entire lungs were dissected and mechanically homogenized using a tissue homogenizer. The homogenates were spun down, and the supernatants were stored at −70 °C. The lung virus titers were determined by using a focus-forming assay (FFA) as described previously [55] with minor modifications. The supernatants of the lung tissue homogenates were serially diluted in DMEM supplemented with 0.1% BSA and N-acetylated trypsin (NAT; 2.5 µg/ml) and added to MDCK cell monolayers. After 1 h of incubation, the cells were overlaid with DMEM supplemented with 1% Avicel, 0.1% BSA and NAT (2.5 µg/ml). After 24 h, the cells were fixed with 4% PFA and incubated with quenching solution (0.5% Triton X-100, 20 mM glycine in PBS). The cells were then treated with blocking buffer (1% BSA, 0.5% Tween-20 in PBS). Focus-forming spots were identified using primary polyclonal goat anti-H1N1 IgG (Virostat, Westbrook, ME, USA), secondary polyclonal rabbit anti-goat IgG conjugated with horseradish peroxidase and TrueBlue^TM^ peroxidase substrate (KPL TrueBlue™, SeraCare Life Science Inc., Milford, MA, USA). The viral titers were calculated as focus-forming units (FFU) per ml of lung tissue homogenate.

### Quantification of T-cell responses

Peripheral blood was harvested, and lymphocytes were isolated as described previously (Oduro et al., 2016). PBMCs were stimulated with peptide at 1 µg/ml in 85 µl of RPMI 1640 for 1 h at 37 °C. Then, 15 μl of brefeldin A (10 µg/ml) was added, and the cells were incubated for an additional 5 h at 37 °C or stained with VNF-specific tetramers (Kindly provided by Ramon Arens, Leiden University) at RT for 30 min. Lymphocytes were stained with fluorescently labeled antibodies against CD3 (17A2, eBiosciences, San Diego, CA, USA), CD4 (GK1.5, BioLegend, San Diego, CA, USA), CD8a (53-6.7, BioLegend, San Diego, CA, USA), CD44 (IM7, BioLegend, San Diego, CA, USA) and CD11a (2D7, Bioscience, San Diego, CA, USA). Subsequently, the cells were fixed and permeabilized (IC fixation buffer and permeabilization buffer, eBioscience, San Diego, CA, USA), and intracellular cytokines were labeled with anti-IFNγ (XMG1.2, BioLegend, San Diego, CA, USA) and anti-TNFα (MP6-XT22, BioLegend, San Diego, CA, USA) antibodies. The labeled cells were analyzed by flow cytometry, and the antigen-specific CD8 + T-cell response was measured.

### Western blot analysis

Sucrose cushion-purified viruses were diluted with PBS, and the protein amounts were then measured by BCA assay according to the manufacturer’s protocol (Pierce™ Micro BCA™ Protein-Assay, Thermo Fisher, Waltham, MA, USA). Samples were treated with 2-mercaptoethanol and sample reducing buffer and then incubated at 95 °C for 5 min. The proteins were separated by SDS–PAGE, transferred to an Immobilon-P PVDF membrane (Millipore Sigma, Munich, Germany) and blocked with 5% milk in TBS-T. Primary antibodies were allowed to bind overnight at 4 °C, after which the membranes were washed in TBS-T and incubated with secondary antibodies for an hour at RT. Upon another wash, images were acquired by a Chemostar PC ECL & Fluorescence Imager (Intas Science, Goettingen, Germany). Anti-SARS-CoV-2 spike (1A9, GeneTex, Irvine, CA, USA), anti-HA (kindly provided by W. Gerhard from Philadelphia), anti-MCK-2 (kindly provided by Stipan Jonjic) and IE1 (IE 1.01, CapRi, Rijeka, Croatia) were used as primary antibodies. Anti-rabbit IgG (ab205718, Abcam, UK) and anti-mouse IgG (ab97046, Abcam, UK) were used as secondary antibodies.

### Immunofluorescence labeling

M2-10B4 cells were infected in a 96-well plate with MCMV^S^ at an MOI of 0.1 for 1 h, washed and incubated for 48 h at 37 °C. Thereupon, the cells were fixed with 4% PFA and incubated with 2% BSA in PBS to block nonspecific binding. Then, the cells were stained with anti-SARS-CoV-2 Spike RBD antibodies (YU519-A09, Yumab) at a 1:200 dilution and anti-GFP (ab13970, Abcam) at a 1:5000 dilution for an hour at RT. After washing three times with PBS, the cells were incubated with secondary antibody mixtures (4410, CST and ab150169, Abcam; 1:800 dilution per antibody) and DAPI at a 1:8000 dilution for an hour at RT. The stained cells were then imaged by a ZEISS LSM 980 confocal microscope using a 20x objective.

### Statistics

Comparisons between two groups were performed using the Mann–Whitney U test (two-tailed). One-way ANOVA with Dunnett’s correction was performed for multiple-group analysis. Two-way ANOVA was used to compare multiple groups at multiple time points. Statistical analysis was performed with GraphPad Prism 6-9.

## Results

### Generation of recombinant MCMVs expressing influenza hemagglutinin or the SARS-CoV-2 S protein

**Fig. 1:**
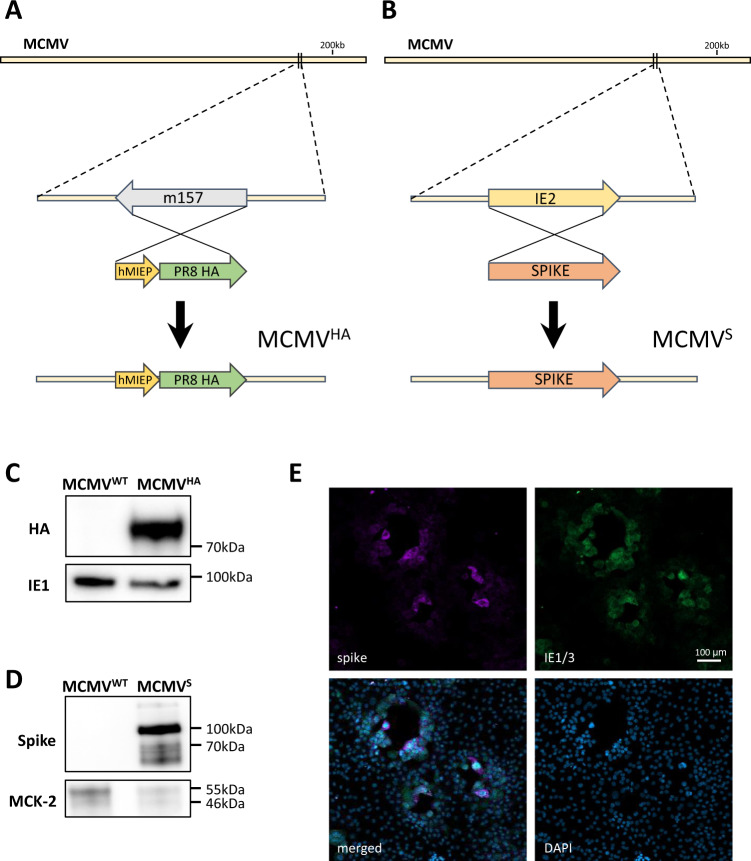
Generation of recombinant MCMV vectors. **A**, **B** Schematic images of the recombinant MCMV vector genome. **A** The HA gene of IAV PR8 was inserted along with minimal hMIEP in the m157 locus. **B** The SARS-CoV-2 spike ORF was inserted in place of ie2. Western blotting of purified virus stocks of MCMV^S^, MCMV^HA^ and MCMV^WT^ was performed with antibodies against (**C**) IAV HA or (**D**) SARS CoV-2 S. As controls, we used (**C**) ie1 or (**D**) MCK-2 proteins of MCMV. **E** M210B4 cells were infected with MCMV^S^ and analyzed at 2 days post-infection (dpi) by immunofluorescence staining of the spike protein to detect its expression or of a GFP protein expressed by the ie1/3 promoter to identify MCMV-infected cells. DAPI staining was used to identify nucleated cells

We recently showed that MCMV^IVL^, a recombinant MCMV vaccine expressing the _**533**_IYSTVASSL_**541**_ epitope (IVL) from the IAV HA protein, protects against influenza challenge when administered intranasally by inducing mucosal-resident CD8^+^ T cells [37]. We hypothesized that recombinant MCMV expressing full-length HA may provide similar or better immune protection. Therefore, we generated a recombinant MCMV expressing full-length HA using a BAC containing the MCMV genome (pSM3fr-MCK-2fl), where the viral m157 gene, which is dispensable for virus in vivo replication [56], was replaced with the whole HA gene (Fig. 1A). Since MCMV vectors expressing ligands for the activating NKG2D receptor show improved immune protection over parental viruses [39], we generated another recombinant MCMV that expressed the Rae-1γ ligand instead of the MCMV gene m152 in addition to the IAV HA gene in the m157 locus and named it Rae-1γMCMV^HA^ (Fig. S1A).

We also generated a recombinant MCMV expressing the gene for the S protein. We replaced the immediate early 2 (ie2) gene of MCMV with the S gene of SARS-CoV-2 because ie2 is dispensable for viral replication and dissemination [57]. Thus, using BAC-based recombination, we generated a new recombinant virus called MCMV^S^ (Fig. 1B). The HA and S proteins were detected by Western blot analysis in the purified virus stocks of MCMV^HA^ or MCMV^S^, respectively (Fig. 1C, D), and exhibited kinetics similar to those of the wild-type virus (not shown). The antibodies used to detect S in WB analysis recognized the S2 domain. To ascertain whether the S protein is expressed as a full-length protein by MCMV^S^, we stained MCMV^S^-infected cells with antibodies against the receptor-binding domain of the S1 domain. We observed that S was mainly localized on the cell surface of MCMV^S^-infected cells (Fig. 1E). In conclusion, our data indicated that MCMV might express full-length membrane-bound antigens from respiratory pathogens.

### Immunization with MCMV expressing the full-length S protein induces neutralizing antibody responses

**Fig. 2:**
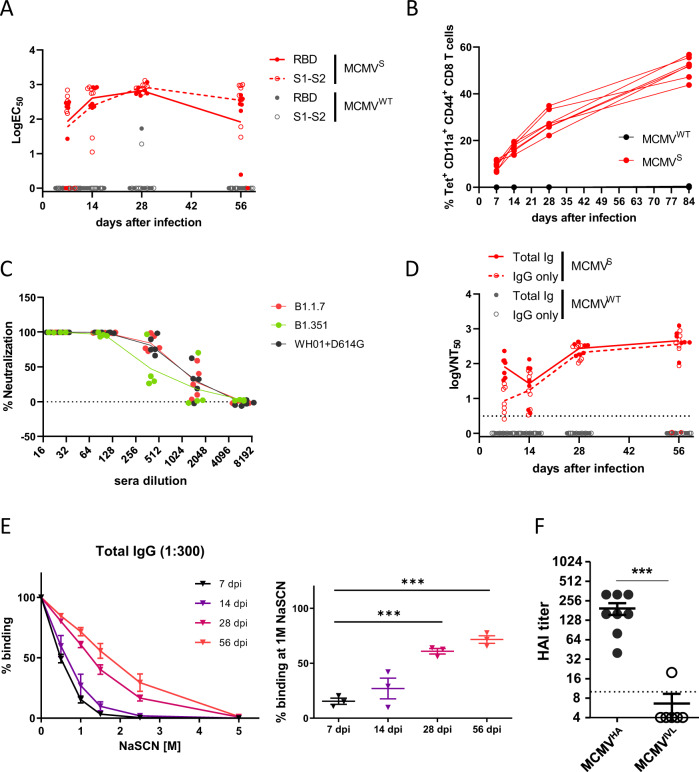
Immunization with MCMV^S^ and MCMV^HA^ elicits antigen-specific and neutralizing humoral responses. BALB/c (**A**, **C**–**E**) or C57BL/6 (**B**) mice were infected i.p. with 2 × 10^5^ PFU or 1 × 10^6^ PFU, respectively, of MCMV^S^ or MCMV^WT^. Collected sera were tested for antigen-specific responses and neutralization capacity. **A** EC_50_ values of IgG isotypes specific for S1-S2 or the RBD. Each symbol indicates an individual mouse, and the geometric means of individual time points are connected with lines. **B** Blood CD8^+^ T cells were analyzed by tetramer staining for the frequency of antigen-specific CD8^+^ T cells in the primed (CD11a^+^CD44^+^) subset. Each line indicates CD8^+^ T-cell responses from one mouse over time. **C** Pseudovirus neutralization capacity (pVNT_50_) against the SARS-CoV-2 S WH01 + D614G, B.1.1.7 and B.1.351 variants. The assay was performed in technical triplicates, and the mean values for each mouse serum sample are shown, where each symbol represents the percentage of neutralization of a mouse serum sample at 56 dpi. The values were calculated as luciferase units. Lines connect the group averages for each serum dilution step. **D** Neutralization capacity (VNT_50_) against SARS-CoV-2 of total serum immunoglobulins (total Igs) or their IgG fraction (IgG only) at the indicated time points post immunization. Each symbol indicates the serum from one mouse, and lines connect the geometric means for each time point. **E** Percentage of serum antibodies binding to the S protein (y axis) in the presence of increasing concentrations of NaSCN (left) or 1 M NaSCN (right) and normalized to the ELISA values in the absence of NaSCN. Each line connects the average residual binding at the indicated molar concentrations of NaSCN. The error bars are standard deviations (SDs). The assay was performed in biological triplicates, and data from the indicated time points post-immunization were statistically compared by one-way ANOVA. *** *p* < 0.001 (**F**) BALB/c mice were i.p. infected with 2 × 10^5^ PFU of indicated virus. The titers of HA-specific antibodies at 28 dpi with MCMV^HA^ or MCMV^IVL^ were tested. The data were pooled from two independent experiments. The long horizontal lines indicate the means. The Mann–Whitney U test was used for statistical analysis. *** *p* < 0.001

BALB/c mice were i.p. immunized with MCMV^S^, MCMV^WT^, or PBS (mock). Blood was collected at 7, 14, 28 and 56 dpi, and sera were tested for antigen-specific immunoglobulin G (IgG) responses against the entire S protein or the receptor binding domain (RBD) by ELISA. We observed notable serum responses in mice immunized with MCMV^S^ at all indicated time points, peaking by 28 dpi (Fig. 2A). To test whether the same vector can elicit cellular immune responses, C57BL/6 mice were immunized with MCMV^S^ or MCMV^WT^, and blood CD8^+^ T-cell responses were monitored by tetramer staining and flow cytometry for the K^b^-restricted VNFNFNGL peptide derived from the S protein [58]. Peptide-specific CD8^+^ T-cell responses were readily detected in MCMV^S^-infected mice but not in MCMV^WT^-infected mice (Fig. 2B). Therefore, MCMV^S^ was capable of eliciting both humoral and cellular immune responses.

We next tested the serum neutralization capacity. We used recombinant vesicular stomatitis virus (VSV) expressing the S gene of SARS CoV-2 isolate hCoV-19/Wuhan/WH01/2019 (B lineage, with introduced D614G mutation), hCoV-19/England/MILK-9E05B3/2020 (B.1.1.7 lineage) or hCoV-19/South Africa/NHLS-UCT-GS-1067/2020 (B.1.351 lineage). Following SARS-CoV-2 S protein-driven cell entry, the pseudoviruses expressed firefly luciferase, which was used as an indicator of infectivity and to analyze the neutralization capacity of the mouse sera. We tested five mouse serum samples collected 56 days post-MCMV^S^ inoculation and observed an average pseudovirus neutralization titer (pVNT_50_) of 1:900 for pseudotypes bearing either SARS-CoV-2 S WH01 + D614G or B.1.1.7 and a slightly reduced but still robust pVNT_50_ of 1:450 for particles harboring SARS-CoV-2 S B.1.351 (Fig. 2C). Therefore, we concluded that the MCMV vector induces robust neutralization titers against multiple clinically relevant SARS-CoV-2 variants. Pseudotyped virus neutralization could be assessed for only one time point due to historical reasons. Hence, we next tested the dynamics of serum neutralization capacity using a bona fide SARS-CoV-2 isolate. Sera were incubated with SARS-CoV-2, and the serum titers resulting in a 50% neutralization of virus (VNT_50_) were determined. A portion of each serum sample was preincubated with 2-mercaptoethanol (2-ME), which specifically destroys the neutralizing activity of IgM [59]. Therefore, these samples essentially showed the neutralization capacity of the IgG antibody class, which was dominant in the serum. We observed that the neutralizing antibody titers against SARS-CoV-2 increased from an average VNT_50_ of 1:84 at 7 dpi to 1:476 at 56 dpi (Fig. 2D and Table 1) for the whole serum fraction and from values below the limit of detection at 7 dpi to 1:407 at 56 dpi for the IgG serum fraction. While 3 out of 29 serum samples (2 samples at 14 dpi and 1 at 56 dpi) did not show any neutralizing capacity (we assume that these mice were not properly immunized due to technical reasons), the vast majority of MCMV^S^-treated mice showed a clear immunization effect (Fig. 2D and S2). On the other hand, MCMV^WT^- and mock-immunized mice showed no specific immune responses against SARS-CoV-2 at any time, suggesting that the protection was specifically induced by the expression of the S protein from the MCMV vector (Fig. S3A, S3B). To verify that neutralizing responses are not restricted to the BALB/c background, we also tested C57BL/6 mice immunized with MCMV^S^ and observed a robust and lasting immune response (Fig. S3C).

**Table 1:** VNT_50_ values of MCMV^S-^immunized mouse serum samples against SARS-CoV-2 or pseudotyped VSV-S

Neutralization (pVNT50 and VNT50)					
	Pseudotyped VSV-S			SARS-CoV-2 (FI strain)	
	WH01			MCMV^S^	MCMV^S^
	+D614G	B1.1.7	B1.351	(Total Ig isotypes)	(IgG isotypes)
day 7				1:84.09	1:10.70
				(33.69–231.6)	(2.54–20.83)
day 14				1:55.93	1:31.90
				(23.36–133.4)	(13.37–63.53)
day 28				1:227.5	1:202.5
				(168–429.5)	(104.7–332.2)
day 56	1:910.5	1:945.2	1:450.6	1:476.4	1:407
				(399.2–1251)	(275.7–883.3)

The table indicates the average VNT_50_ or pVNT_50_ values against SARS-CoV-2 or pseudotyped VSV-S of serum samples collected from immunized mice at the indicated time points. The neutralizing antibody titers were calculated by nonlinear IC50 regression analysis in GraphPad Prism 9. The indicated VNT_50_ or pVNT_50_ values denote the serum dilutions that resulted in a 50% reduction of virus plaques or of VSV luciferase activity.

Interestingly, the neutralization titers in the 2-ME-treated groups were essentially comparable to those in the untreated groups by 28 dpi (Fig. 2D and Table 1), suggesting that the fraction of class-switched antibodies increased significantly at later time points. Furthermore, the antibody titer peaked at 28 dpi, while the neutralization capacity continued to expand until the last measured time point at 56 dpi. Taken together, these observations implied a potential germinal center reaction leading to somatic hypermutations and affinity maturation. We therefore measured the binding avidity of serum antibodies over time by sodium thiocyanate (NaSCN) inhibition [50, 60]. Sera from MCMV^S^-immunized mice were treated with graded amounts of NaSCN, and residual binding to S was determined by ELISA as an indicator of avidity. Binding avidity increased consistently and continuously from 7 to 56 dpi (Fig. 2E). Therefore, the increase in neutralization capacity over time (Fig. 2D) was matched by an increase in binding avidity (Fig. 2E) rather than by an increase in the amount of antibody (Fig. 2A).

### Immunization with MCMV expressing the full-length HA protein induces neutralizing antibody responses

To test whether antibody responses can be elicited against another respiratory virus, we tested the hemagglutination inhibition (HAI) serum titers at 28 days post-immunization with MCMV^HA^. As a control, we used MCMV^IVL^, an MCMV expressing solely an immunodominant MHC-I-restricted octameric epitope from IAV HA [37]. HA-specific antibodies were detected in MCMV^HA^-immunized mice but not in those vaccinated with MCMV^IVL^ (Fig. 2F). Therefore, the MCMV vaccine vector expressing the full-length HA induced humoral responses that recognized HA and impaired its binding, and this was not due to cross-reactivity to MCMV antigens, as seen in the MCMV^IVL^ control group.

### MCMV^HA^ vaccination induces robust immune protection but weak CD8^+^ T-cell responses

**Fig. 3:**
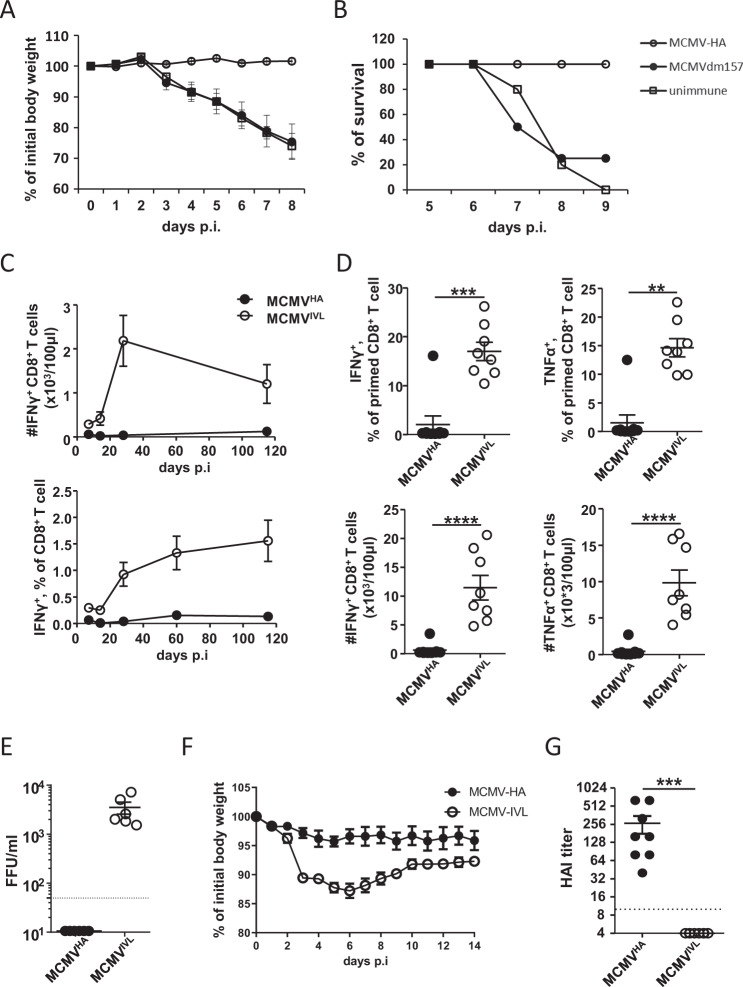
MCMVHA immunization induces robust immune protection but a poor IVL-specific CD8^+^ T-cell response. **A**, **B** C57BL/6 female mice were immunized f.p. with 2 × 10^5^ PFU of MCMV^HA^ or MCMV^Δm157^ virus. After 21 days, the mice were challenged with IAV (100 HU). **A** Body weight loss and (**B**) survival rates were measured. **C**–**G** BALB/c mice were infected i.p. with 2 × 10^5^ PFU of MCMV^HA^ or MCMV^IVL^. **C** Blood leukocytes were isolated and stimulated in vitro with IVL peptide for 6 h and analyzed by intracellular cytokine staining (ICCS) by flow cytometry at the indicated dpi. The percentages and numbers of IVL-specific CD8^+^ T cells in peripheral blood are shown as averages ± SDs (*n* = 6–10). **D**–**G** Immunized mice were challenged with IAV (1100 FFU, i.n.) at > 3 months p.i. Blood leukocytes were stimulated in vitro with IVL peptide for 6 h and analyzed by ICCS by flow cytometry on Day 7 post-challenge. Each symbol represents an individual mouse sample. (E) IAV titers measured by focus-forming assay (FFA) in the lungs on Day 5 post-challenge. Each symbol represents an individual mouse. **F** Average body weight loss upon IAV challenge at the indicated time points (*n* = 6–9). **G** Titers of HA-specific antibodies in serum samples on Day 5 after IAV challenge. The titers were detected by HA inhibition assay, *n* = 6–9. Each symbol represents an individual mouse. All of the above data are pooled data from two independent experiments. The Mann–Whitney U test was used for statistical analysis. ** *p* < 0.01, *** *p* < 0.001, **** *p* < 0.0001

C57BL/6 mice were i.p. immunized with MCMV^HA^. Control groups were not infected or infected with the parental virus MCMV^Δm157^, which lacks the *m157* gene but does not express the HA gene. The mice were challenged with lethal IAV doses at 21 days post-immunization, and weight loss and mortality were followed. PBS-immunized mice showed severe body weight loss and mortality, whereas MCMV^HA^-immunized mice showed no weight loss and no mortality (Fig. 3A and B). We compared the effects of immunization with Rae-1γMCMV^HA^ to the effects of immunization with parental MCMV^HA^ and MCMV^Δm157^ viruses by measuring weight loss kinetics for 5 days upon IAV challenge. We also measured flu virus titers in the lungs of challenged mice at 5 days post-IAV challenge. While weight loss was averted in both groups immunized with MCMVs expressing the HA gene, and while flu titers were reduced in the same groups relative to the control groups that were immunized with MCMV^Δm157^ or not immunized at all (Fig. S1B and S1C), Rae-1γMCMV^HA^ immunization did not reduce titers more efficiently than MCMV^HA^. Hence, the MCMVs encoding HA provided immune protection against IAV challenge irrespective of RAE1γ expression.

Since MCMV^HA^ provided immune protection even when applied i.p., whereas MCMV^IVL^ was protective only when applied i.n. [37], and since HAI titers were induced exclusively by MCMV^HA^ (Fig. 2F), we hypothesized that MCMV^HA^ induces stronger adaptive immune responses than MCMV^IVL^. While intraperitoneal administration of MCMV^IVL^ elicits powerful responses to the HA peptide over an essentially negative background induced by MCMV-wt infection [37], we could not exclude the possibility that MCMV^HA^ may induce even stronger CD8^+^ responses. Therefore, we compared IVL-specific K^d^-restricted CD8^+^ T-cell responses upon MCMV^IVL^ or MCMV^HA^ infection in BALB/c mice. We monitored the frequency of IFNγ^+^ CD8^+^ T cells until 115 dpi and observed that IFNγ^+^ CD8^+^ T-cell counts were robustly increased in MCMV^IVL^-immunized mice but barely increased in MCMV^HA^-immunized mice in both relative and absolute terms (Fig. 3C). Similar responses were observed at 7 days post-IAV challenge: mice primed with MCMV^IVL^ showed substantially strengthened cytokine responses in the CD8^+^ compartment upon in vitro restimulation with the IVL peptide (Fig. 3D). Hence, our data indicated that the superior protection provided by MCMV^HA^ may not be due to enhanced cellular immunity.

To directly compare the protective capacity of MCMV^IVL^ and MCMV^HA^, we challenged the mice introduced in Fig. 2F with IAV at times of virus latency (>3 months post-immunization) and measured the IAV titers in the lungs of immunized mice at 5 days post-challenge. No infectious IAV could be detected in MCMV^HA^-immunized mice, whereas all MCMV^IVL^-immunized mice showed clearly detectable virus titers (Fig. 3E). We also monitored body weight upon IAV challenge and observed a significant drop in mice immunized with MCMV^IVL^ but not in those immunized with MCMV^HA^ (Fig. 3F). Intriguingly, only MCMV^HA^-immunized mice showed robust HAI serum titers at 5 days post-IAV challenge (Fig. 3G). Therefore, our results suggest that the virus expressing the full-length HA gene provides better immune protection than the other virus, likely due to humoral immune responses.

### The MCMV full-length-protein vector protects against viral challenge through a humoral response

**Fig. 4:**
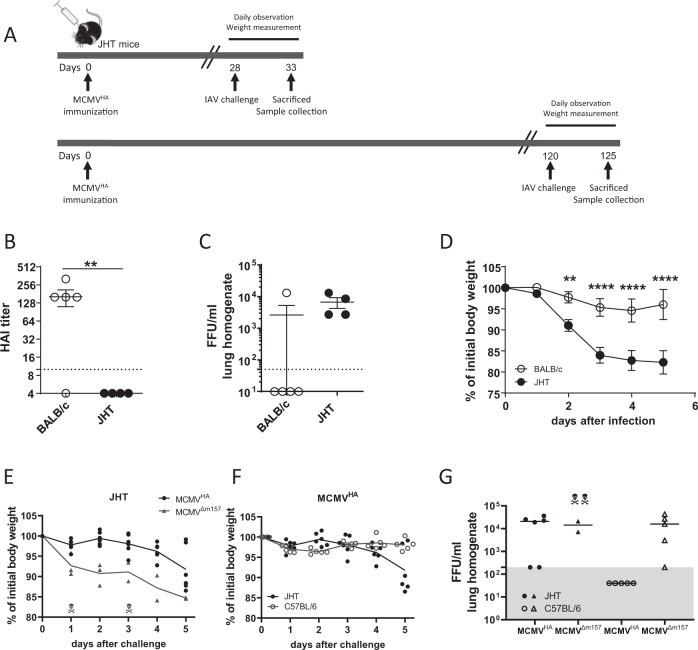
Humoral immunity elicited by MCMVHA protects against IAV challenge. JHT and BALB/c mice (**A**–**D**) or C57BL/6 mice (**A**, **E**–**G**) were immunized i.p. with 2 × 10^5^ PFU of MCMV^HA^. Twenty-eight days (**A**, **E**–**G**) or 120 days (**A**–**D**) after immunization, the mice were challenged with IAV (1100 FFU, i.n.). **A** Setup of the experiment. **B** Titers of HA-specific antibody in serum samples on Day 5 after IAV challenge determined by HAI. **C** IAV lung titers on Day 5 post-challenge. **D** Body weight loss upon IAV challenge. All data are pooled data from two independent experiments. **E** Body weight loss upon IAV challenge of MCMV^HA^- or control-immunized (MCMV^∆m157^) JHT mice. **F** Comparison of body weight loss of MCMV^HA^-immunized JHT and control mice. **G** Titers of IAV in the lungs on Day 5 post-challenge. Each symbol represents an individual mouse sample, and the skull-and-crossbones symbols indicate dead animals. The horizontal lines indicate the means, and the error bars indicate the standard error values. Two-way ANOVA (for Fig. 4D) or Mann–Whitney U tests (for Fig. 4B and C) were used for statistical analysis. ** *p* < 0.01, *** *p* < 0.001, **** *p* < 0.0001

While our results showed that MCMVs expressing viral glycoproteins induce neutralizing immunity, it was not formally proven that humoral immunity was essential for immune protection upon challenge. To test this hypothesis directly, we immunized B-cell-deficient JHT mice with MCMV^HA^ and challenged them with IAV at 28 or 120 days post-immunization (Fig. 4A). As expected, HA-specific antibodies were observed only in the immunized BALB/c mice, while no functional HA-specific antibodies could be detected in JHT mice (Fig. 4B). Similarly, IAV challenge at 120 dpi resulted in viral titers that were detected only in the lungs of vaccinated JHT mice but were absent from BALB/c controls (Fig. 4C). Moreover, JHT mice suffered significant weight loss upon IAV challenge, whereas BALB/c mice did not (Fig. 4D). To define whether MCMV^HA^ protection also occurs at early time points and to identify any B-cell-independent protection in JHT mice, we immunized JHT mice or littermate controls with MCMV^HA^ or mock-immunized them with MCMV^Δm157^ and challenged all of them with IAV at 28 dpi. Most JHT mice lost weight upon challenge, but the effect was more pronounced in MCMV^Δm157^-immunized mice; some of these mice succumbed to the infection prior to organ harvest at 5 dpi (Fig. 4E). On the other hand, no weight loss was observed in MCMV^HA^-immunized littermates (Fig. 4F). Likewise, MCMV^HA^-immunized littermates completely controlled IAV replication upon challenge, whereas infectious virus was detected in the majority of MCMV^HA^-immunized JHT mice (Fig. 4G). Taken together, these results indicated a critical role of antibodies in controlling IAV. Our data demonstrate that MCMVs expressing a full-length protein provide immune protection against respiratory viral challenge and that this protection depends on the humoral response to neutralizing antibodies.

## Discussion

CMV has aroused great interest as a vaccine vector in recent years due to its strong immunogenicity and ability to establish a life-long inflationary CD8^+^ T-cell response [61]. Many studies have demonstrated that exogenous antigens fused to the CMV genome provide protection against corresponding pathogens, but almost all of the previous publications have focused on T-cell-based immune protection [17, 18, 20, 21, 28, 37] and barely covered B-cell-based humoral responses, which prevent viral spread via extracellular fluids [62]. One detailed study has shown that MCMVs induce protective humoral immune responses against a murine retrovirus [63], but it has remained unclear if this principle applies to clinically relevant pathogens. Humoral immunity against the Ebola glycoprotein protein was observed upon immunization of rhesus monkeys with an RhCMV vector by ELISA, but the sera showed no virus neutralization capacity [21]. In this study, we constructed MCMV-based vaccine vectors against two pandemic viruses, IAV and SARS-CoV-2, and demonstrated that neutralizing humoral immune responses against both were induced by recombinant MCMV vaccination and that immune protection was abrogated in B-cell-deficient mice. Humoral immunity elicited by MCMV^HA^ provided better protection against IAV challenge than the robust cellular immunity elicited by MCMV^IVL^. Furthermore, the insertion of Rae-1γ was previously shown to promote memory CD8^+^ T-cell responses, thus improving protection by CMV vectors [39, 64, 65], but Rae-1γ did not improve MCMV^HA^ protection against IAV challenge in this system, arguing against a role of T cells in MCMV^HA^-mediated protection. Overall, our evidence strongly suggests that humoral immunity is both sufficient and necessary to provide immune protection against respiratory virus challenge. A limitation of our study is that we did not investigate CD4 T-cell responses in MCMV^HA^-immunized mice. Another limitation is that the lack of immune protection in JHT mice might have also been a result of the impaired activity of antigen-presenting cells that stimulate CD4 T cells. We consider this a remote possibility, as other antigen-presenting cells were still available in JHT mice. Therefore, a conclusive set of data excluding CD4 contributions to the observed phenotypes needs to be generated in future studies, although the role of CD4 appears to be minor. Another limitation was the assessment of cross-neutralizing activity, which was performed at a single late time point. In future studies, we will analyze the kinetics of cross-neutralizing responses induced by MCMV vectors.

Previous results have shown, however, that MCMV vectors expressing a single MHC-I restricted peptide are sufficient to provide protection against viral challenge [20, 28, 33, 35]. Even more surprisingly, others have shown that MCMVs expressing a single immunodominant peptide provide better protection than those carrying the full-length protein [34, 66]. Here, we observed the exact opposite phenotype: the full-length protein provided better protection. However, systemic inflationary T-cell responses do not protect against influenza or respiratory syncytial virus [37, 44], and intraperitoneal immunization, as performed in this study, does not elicit protective lung mucosal CD8^+^ T-cell responses. Therefore, our data may indicate that respiratory viral infections may be controlled better by systemic humoral immunity than by systemic cellular immunity.

Our study did not address the potential of CMV vectors to elicit mucosal humoral immunity and whether they provide enhanced protection against IAV or SARS-CoV-2 infection. This question is intriguing but goes beyond the scope of the present manuscript. Hence, it needs to be addressed in future studies whether intranasal administration of MCMV vectors induces mucosal (IgA) antibody responses and whether this further improves immune protection.

Immediate early genes, especially ie1 and ie2 of MCMV, are sporadically expressed during latency [67], enhancing memory inflation [68]. A similar induction of humoral immunity against CMV antigens has also been documented [50]. We used promoters to express the S or HA proteins and observed an ongoing increase in the neutralizing capacity of sera up to 8 weeks post-infection and robust immune protection at three months post-immunization. There remains a clinical need for vaccines eliciting long-term immune protection and obviating the need for booster responses, and the gap might be closed by CMV-based formulations. One explanation for the long-term immunity may be the continuous restimulation of antigen-specific B cells by sparse antigen expression during latency, which boosts B-cell immunity over time. While this hypothesis needs to be experimentally validated in future studies, our data demonstrated that the levels of neutralizing antibodies increased over time, that the levels of class-switched isotypes gradually increased and dominated at later time points after vaccination and that this was concomitant with an increase in avidity. All of these findings implicate somatic hypermutation processes in germinal center reactions elicited by MCMV vector immunization.

In summary, our data suggest that a single injection of an MCMV vector may be sufficient to induce protective B-cell memory responses against respiratory viral pathogens. This effect is an improvement over those of most of the currently available vaccine formulations against COVID-19. Our data also suggest that CMV vectors might be useful as vaccine tools against other pathogens that may emerge in the future. In light of observations that replication-deficient CMV vectors provide long-term immune protection [40, 42, 69], it is possible to envisage vaccine formulations that combine safety and long-term humoral immune protection. This study provides crucial insights to support the development of such formulations.

## Supplementary information

Supplementary figures

Supplementary figure legends
