# Supplementary figures and images for: MCMV-based vaccine vectors expressing full-length viral proteins provide long-term humoral immune protection upon a single-shot vaccination
Source: Cell Mol Immunol. 2022 Jan 7;19(2):234–44. doi: 10.1038/s41423-021-00814-5 (PMC8739032; doi:10.1038/s41423-021-00814-5)

**A**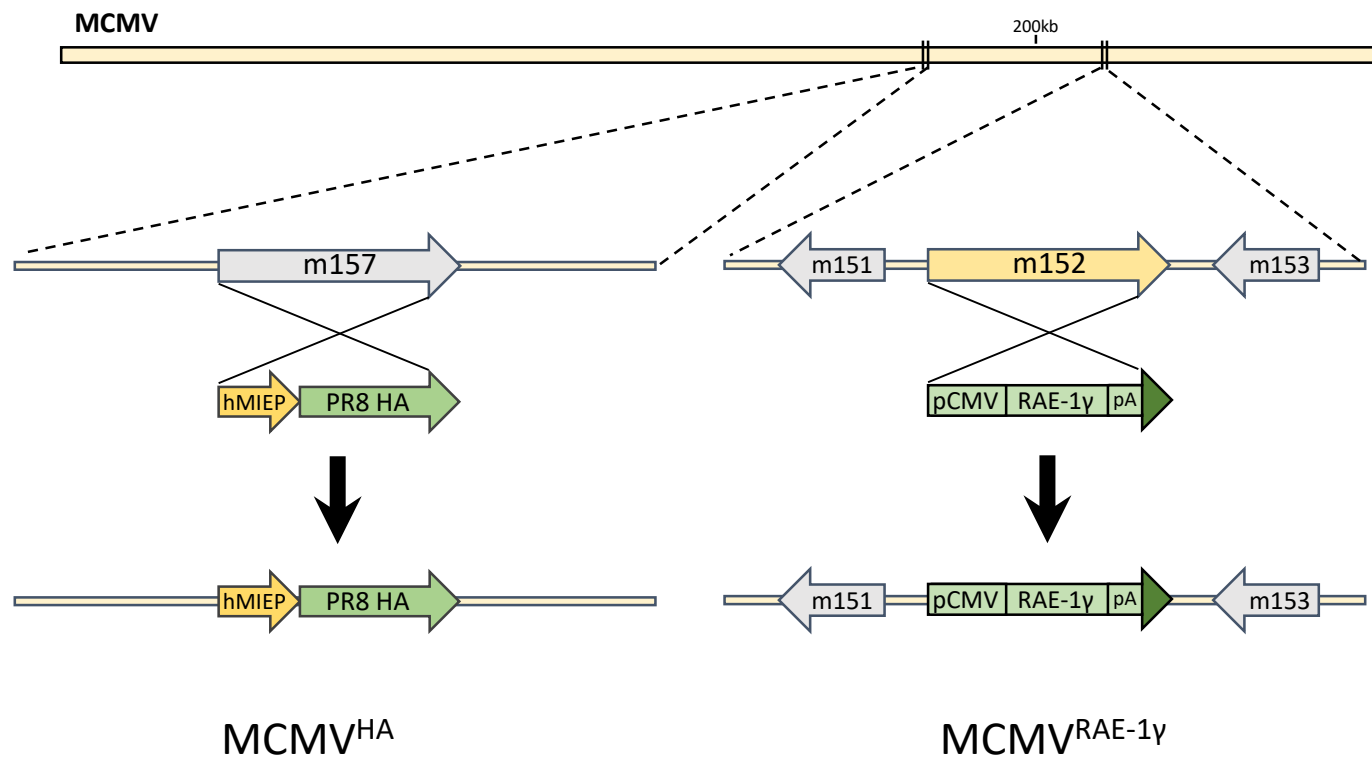**B**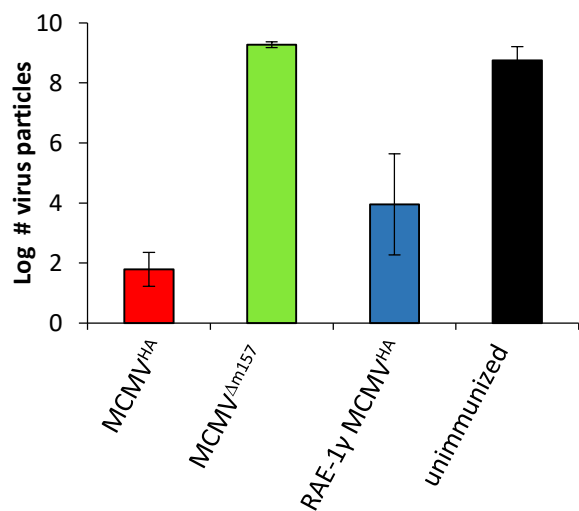**C**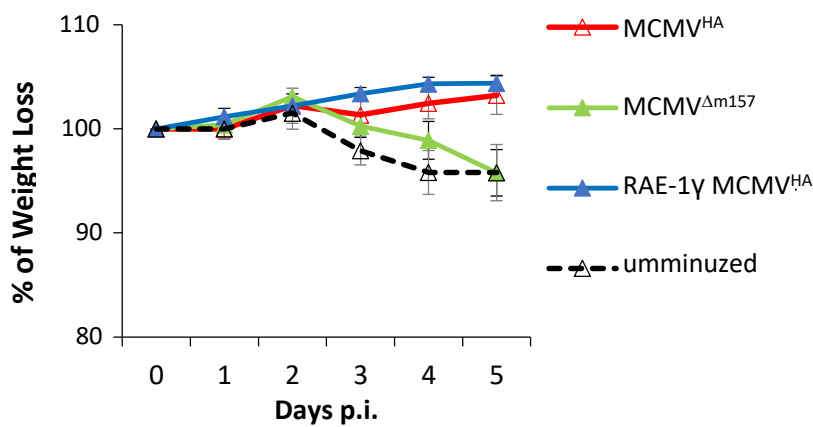

**A**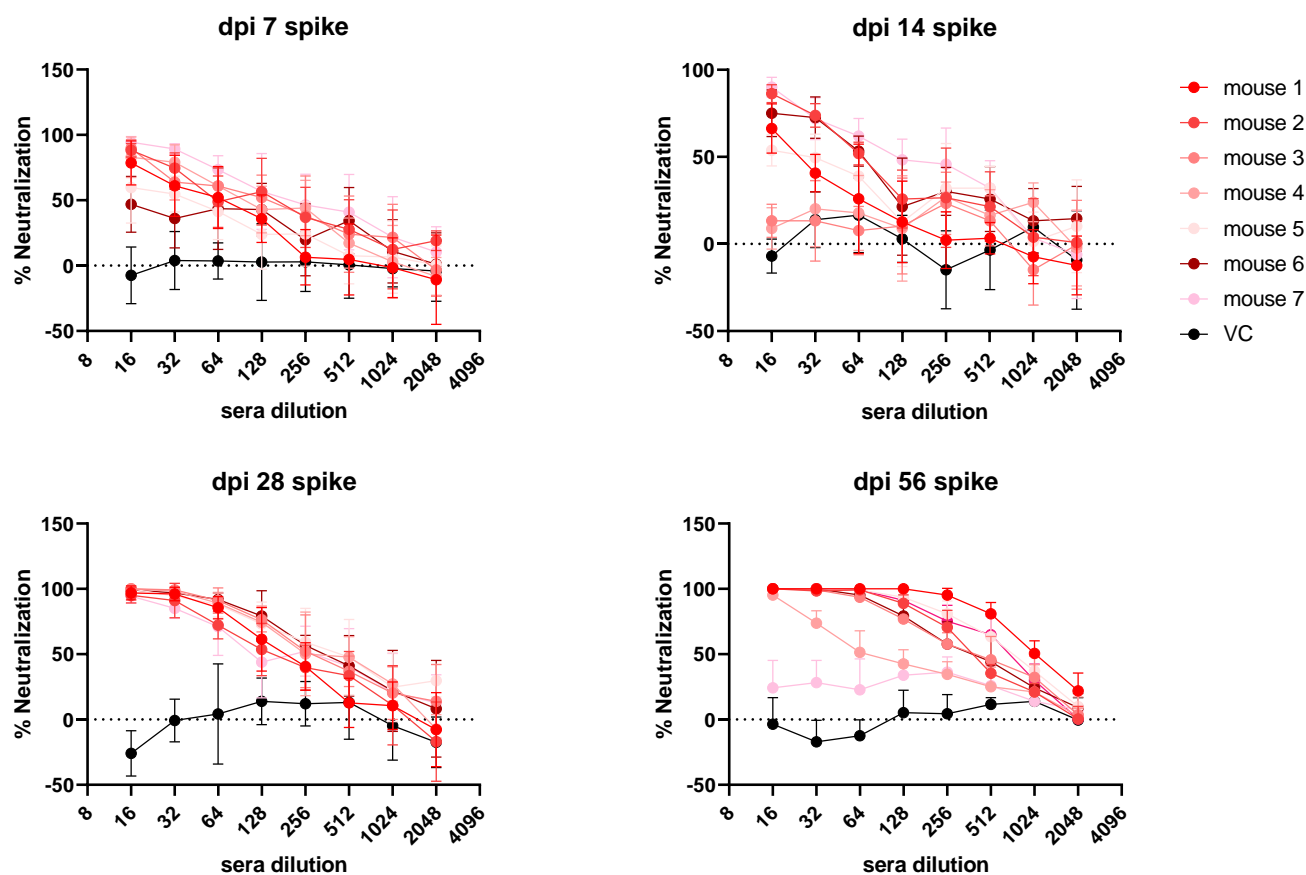**B**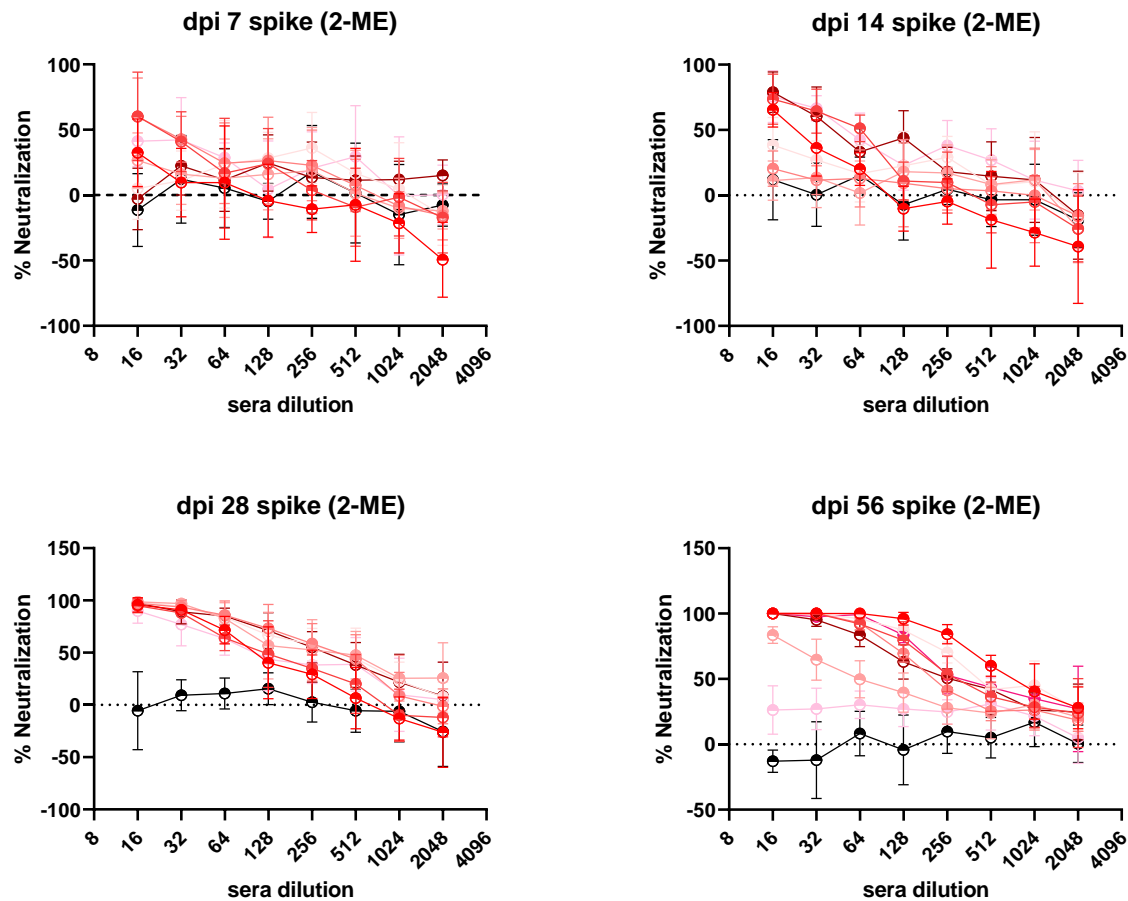

**A****dpi 7 WT**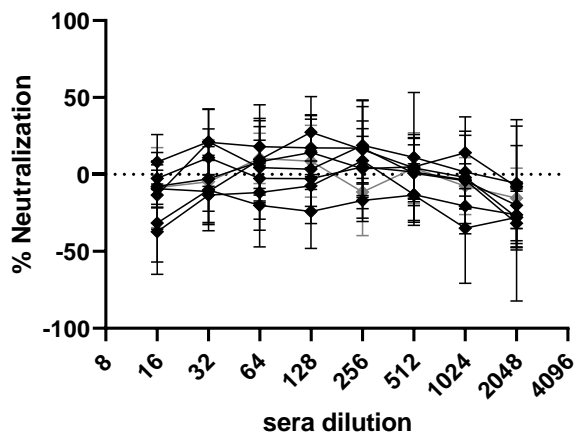**dpi 28 WT**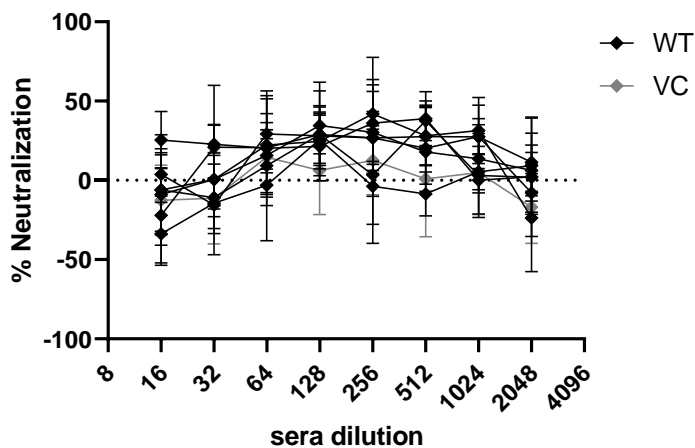**dpi 14 WT**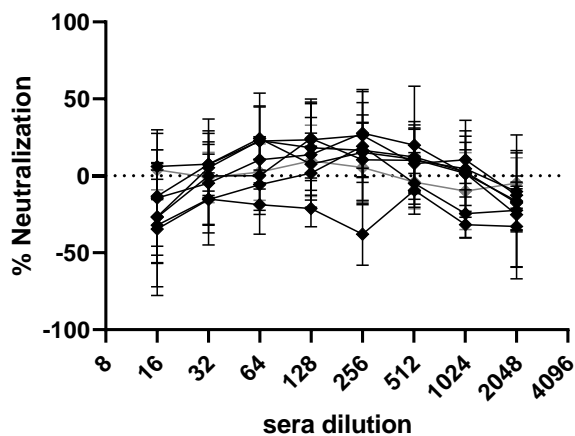**dpi 56 WT**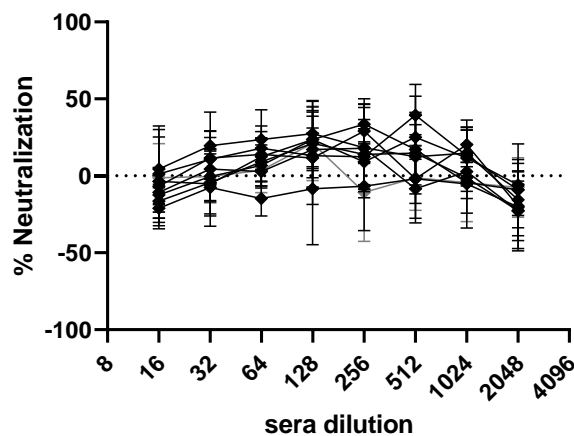**B**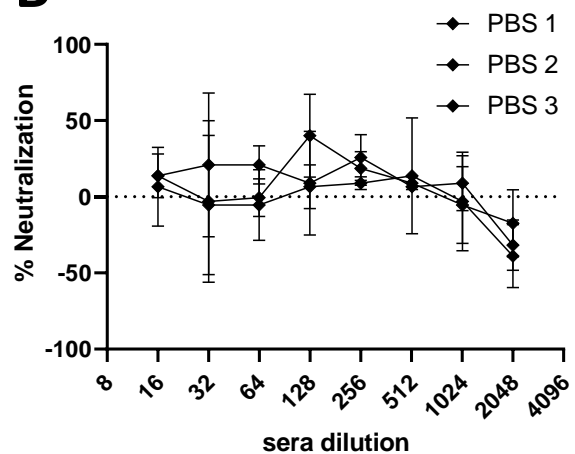**C****C57BL/6**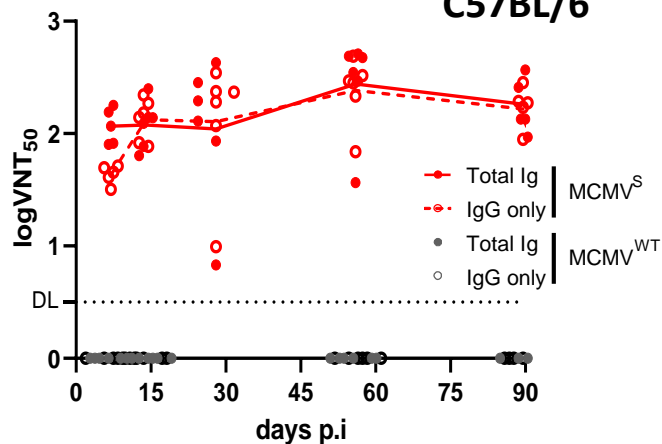

Supplement: Supplementary file 1 — Supplementary figures [file 41423_2021_814_MOESM1_ESM.pdf]
