# Supplementary material for: MCMV-based vaccine vectors expressing full-length viral proteins provide long-term humoral immune protection upon a single-shot vaccination
Source: Cell Mol Immunol. 2022 Jan 7;19(2):234–44. doi: 10.1038/s41423-021-00814-5 (PMC8739032; doi:10.1038/s41423-021-00814-5)
Supplement: Supplementary file 2 — Supplementary figure legends [file 41423_2021_814_MOESM2_ESM.pdf]

Supplementary figure legends

Figure S1. Characterization of Rae1γ-MCMV^{HA}

(A) Schematic representation of the Rae1γ-MCMV^{HA} genetic construct. (B) Mice were immunized with indicated MCMV vectors and challenged with IAV. Infectious virus titers in organs was measured and medians and ranges are shown. (C) Weight loss kinetics upon IAV challenge of mice immunized with indicated MCMV vectors. Means are connected by lines, error bars are SD.

Figure S2. Neutralization titers of sera from MCMV^{S} immunized mice

BALB/c female mice were immunized with 2x10^{5} PFU MCMV^{S} virus via the i.p. route. At indicated dpi, virus neutralization titers of sera from heart blood were measured. Neutralization of (A) total serum isotypes or (B) the IgM-depleted fraction against SARS-CoV-2 are shown. Each line indicates the serum dilution series from one mouse sample. Neutralization efficacy was calculated as the difference between the number of plaques observed at each dilution step and the average number of plaques from control wells infected in absence of sera, normalized to a 100% threshold. Red lines correspond to titrations in presence of sera from MCMV^{S} immunized mice, the black line is the virus control in absence of diluted sera. VC – virus control (virus infected cells in absence of sera).

Figure S3. Neutralization curve of MCMV^{WT} immunized mouse serum

(A) BALB/c female mice were mock-immunized with 2x10^{5} PFU MCMV^{WT} virus via the i.p. route. At indicated dpi, virus neutralization titers of sera from heart blood were measured as in Figure S2. Each line indicates the serum dilution series from one mouse and error bars are SD. VC – virus control (virus infected cells in absence of sera). (B) BALB/c female mice were mock-immunized with PBS via the i.p. route and sera from day 14 post immunization were analyzed as

in the panel A. (C) C57BL/6 female mice were immunized with 10^6 PFU of $MCMV^S$ or $MCMV^{WT}$ and sera were analyzed for virus neutralization titers in presence of absence of 2-ME at indicated time points (dpi 7, 14, 28, 56 and 90). Each symbol indicates an individual mouse and lines connect geometrical means. The dashed line represents the detection limit.
